# Cryptococcal Meningitis Treatment Strategies in Resource-Limited Settings: A Cost-Effectiveness Analysis

**DOI:** 10.1371/journal.pmed.1001316

**Published:** 2012-09-25

**Authors:** Radha Rajasingham, Melissa A. Rolfes, Kate E. Birkenkamp, David B. Meya, David R. Boulware

**Affiliations:** 1 Infectious Disease Institute, Makerere University, Kampala, Uganda; 2 Division of Infectious Diseases & International Medicine, Department of Medicine, University of Minnesota, Minneapolis, Minnesota, United States of America; Hospital for Tropical Diseases, Viet Nam

## Abstract

**Background:**

Cryptococcal meningitis (CM) is the most common form of meningitis in Africa. World Health Organization guidelines recommend 14-d amphotericin-based induction therapy; however, this is impractical for many resource-limited settings due to cost and intensive monitoring needs. A cost-effectiveness analysis was performed to guide stakeholders with respect to optimal CM treatment within resource limitations.

**Methods and Findings:**

We conducted a decision analysis to estimate the incremental cost-effectiveness ratio (ICER) of six CM induction regimens: fluconazole (800–1,200 mg/d) monotherapy, fluconazole + flucytosine (5FC), short-course amphotericin (7-d) + fluconazole, 14-d of amphotericin alone, amphotericin + fluconazole, and amphotericin + 5FC. We computed actual 2012 healthcare costs in Uganda for medications, supplies, and personnel, and average laboratory costs for three African countries. A systematic review of cryptococcal treatment trials in resource-limited areas summarized 10-wk survival outcomes. We modeled one-year survival based on South African, Ugandan, and Thai CM outcome data, and survival beyond one-year on Ugandan and Thai data. Quality-adjusted life years (QALYs) were determined and used to calculate the cost-effectiveness ratio and ICER. The cost of hospital care ranged from $154 for fluconazole monotherapy to $467 for 14 d of amphotericin + 5FC. Based on 18 studies investigating outcomes for HIV-infected individuals with CM in resource-limited settings, the estimated mean one-year survival was lowest for fluconazole monotherapy, at 40%. The cost-effectiveness ratio ranged from $20 to $44 per QALY. Overall, amphotericin-based regimens had higher costs but better survival. Short-course amphotericin (1 mg/kg/d for 7 d) with fluconazole (1,200 mg/d for14 d) had the best one-year survival (66%) and the most favorable cost-effectiveness ratio, at $20.24/QALY, with an ICER of $15.11 per additional QALY over fluconazole monotherapy. The main limitation of this study is the pooled nature of a systematic review, with a paucity of outcome data with direct comparisons between regimens.

**Conclusions:**

Short-course (7-d) amphotericin induction therapy coupled with high-dose (1,200 mg/d) fluconazole is “very cost effective” per World Health Organization criteria and may be a worthy investment for policy-makers seeking cost-effective clinical outcomes. More head-to-head clinical trials are needed on treatments for this neglected tropical disease.

*Please see later in the article for the Editors' Summary.*

## Abstract 2

David Boulware and colleagues assess the cost effectiveness of different treatment strategies in low- and middle-income countries for cryptococcal meningitis, one of the most common opportunistic infections of people with HIV.

## Editors' Summary

**Background:**

Cryptococcal meningitis, a fungal infection of the membranes around the brain and spinal cord, affects about a million people every year (most of them living in sub-Saharan Africa and Southeast Asia) and kills about 640,000 people annually. People become infected with *Cryptococcus neoformans*, the fungus that causes cryptococcal meningitis and which is found in soil and dirt, by breathing it in. In healthy individuals, infection rarely causes disease. But in people living with AIDS, whose immune system has been damaged by HIV infection, and in people whose immune system is compromised for other reasons, the fungus can invade and damage many organs, including the brain. Cryptococcal meningitis, the symptoms of which include fever, stiff neck, headache, and vomiting, is diagnosed by looking for the fungus in fluid taken from the spinal cord in a procedure called a lumbar puncture. Cryptococcal meningitis is treated with antifungal drugs such as amphotericin, fluconazole, and flucytosine (induction therapy); recurrence of the infection is prevented by taking fluconazole daily for life or until the immune system recovers.

**Why Was This Study Done?:**

The World Health Organization (WHO) recommends a 14-day regimen of intravenous (injected) amphotericin and oral flucytosine or fluconazole for induction therapy of cryptococcal meningitis. Unfortunately, this regimen is impractical in many resource-limited settings because of the cost of the drugs and hospital care and the need for intensive monitoring—amphotericin is extremely toxic. Consequently, high-dose fluconazole monotherapy is the usual treatment for cryptococcal meningitis in resource-limited countries, although this regimen is much less effective. Another regimen that has improved survival in trials is flucytosine with fluconazole for two weeks. However, flucytosine is very expensive and is not licensed in most sub-Saharan African countries. Stakeholders in developing countries badly need guidance, therefore, on which induction treatment for cryptococcal meningitis they should recommend to optimize outcomes in their particular countries. In this cost-effectiveness analysis (a study that compares the costs and health effects of different interventions), the researchers use costs in Uganda to estimate the survival, cost, and cost per benefit associated with various induction treatments for cryptococcal meningitis in HIV-infected patients.

**What Did the Researchers Do and Find?:**

The researchers calculated the overall cost of six induction treatments using 2012 healthcare costs in Uganda for medications, supplies, and hospital care, and average laboratory costs for monitoring treatment from three African countries. They used data from published trials of cryptococcal meningitis treatment in resource-limited areas to estimate ten-week and one-year survival, life expectancy, and quality-adjusted life years (QALYs, the number of years of life added by an intervention, adjusted for the quality of life) for each intervention. Finally, they calculated the cost-effectiveness ratio (cost per QALY gained) and the incremental cost effectiveness ratio (ICER, the additional cost of a treatment strategy compared to fluconazole monotherapy divided by the incremental improvement in QALYs) for each intervention. The estimated costs per person for each induction treatment strategy ranged from US$154 for 14 days of fluconazole monotherapy to US$467 for 14 days of amphotericin plus flucytosine. Estimated average one-year survival was lowest for fluconazole (40%) and highest for short-course (seven days) amphotericin plus 14 days of fluconazole (66%), similar to other amphotericin-based treatments. Cost-effectiveness ratios ranged from US$20 per QALY for short-course amphotericin plus fluconazole to US$44 per QALY for 14 days of amphotericin plus flucytosine. Short-course amphotericin plus fluconazole had the lowest ICER (US$15.11 per additional QALY over fluconazole monotherapy).

**What Do These Findings Mean?:**

These findings suggest that, among the treatments investigated, a seven-day course of amphotericin with high-dose fluconazole for at least two weeks is the most cost-effective induction treatment for cryptococcal meningitis in Uganda. Although this result should be generalizable to other African countries, it needs to be treated with caution because very few trials have actually looked at the clinical effectiveness of this particular regimen. While short short-course amphotericin appears to be substantially more effective than fluconazole monotherapy, large-scale trials comparing short-course amphotericin regimens with more traditional 14-day regimens in resource-limited countries must be undertaken before short-course amphotericin-based treatments are adopted. Notably, however, if these trials confirm that survival with short-course amphotericin with fluconazole is about 30% better than with fluconazole alone, the researchers calculate that moving to short-course amphotericin could save about 150,000 lives every year in sub-Saharan Africa at a cost of US$220 per life saved.

**Additional Information:**

Please access these websites via the online version of this summary at http://dx.doi.org/10.1371/journal.pmed.1001316.

- This study is further discussed in a *PLOS Medicine* Perspective by Andrew Farlow
- Preventcrypto.org provides a clearinghouse for updated guidelines for cryptococcal diagnosis and treatment.
- The US Centers for Disease Control and Prevention provides information on *Cryptococcus neoformans* and a training manual called the Cryptococcal Screening Program Training Manual for Healthcare Providers
- NAM/aidsmap provides information about all aspects of infection with *Cryptococcus neoformans*, including a personal story about cryptococcal meningitis
- AIDS InfoNet has a fact sheet on cryptococcal meningitis (in several languages)
- The not-for-profit organization Project Inform, which provides information, inspiration, and advocacy for people with HIV/AIDS and hepatitis C (in English and Spanish), has a fact sheet on cryptococcal meningitis
- The MedlinePlus encyclopedia has a page on cryptococcal meningitis (in English and Spanish)

## Introduction

Cryptococcal meningitis (CM) affects an estimated 957,900 people per year, with the overwhelming burden of disease in sub-Saharan Africa and Southeast Asia, where annual mortality may equal or exceed that for tuberculosis [1],[2]. The 2011 World Health Organization (WHO) guidelines for the management of CM recommend treatment with amphotericin-based regimens to optimize survival, neurologic outcomes, and fungal clearance [3].

For induction treatment, a 2-wk regimen of intravenous (IV) amphotericin B with oral flucytosine (5FC) is strongly favored given three randomized controlled trials demonstrating increased fungal clearance and reduced risk of relapse compared to amphotericin alone [4]–[7]. Where 5FC is not available, combination therapy of amphotericin + fluconazole (800 mg) daily for 2 wk is the preferred regimen over 2 wk of amphotericin alone [3],[8]. Unfortunately, these recommendations do not account for local availability of medications, costs of care, capacity for hospitalization with intensive monitoring, or management of amphotericin-related toxicities in resource-limited settings, where the prevalence of cryptococcosis remains the highest.

The WHO guidelines recommend that where amphotericin is not feasible, one should consider an oral regimen of high-dose fluconazole (800–1,200 mg/d) with 5FC, if available, while recognizing that standard-dose fluconazole (≤400 mg/d) is merely fungistatic, and that high-dose fluconazole (800–1,200 mg/d) is still significantly less microbiologically effective than amphotericin-based regimens [7],[9],[10]. The combination of 5FC with fluconazole for 2 wk results in decreased mortality compared to fluconazole alone [11],[12]. However, 5FC is remarkably expensive, not licensed in most sub-Saharan African countries, and therefore unavailable. One alternative to the above regimens is 5–7 d of amphotericin alone or in combination with 2 wk of high-dose (1,200 mg) fluconazole [13]. Small observational studies in resource-constrained settings have employed short courses of amphotericin, as it is less expensive and has less toxicity than 2 wk of amphotericin, with greater microbiologic activity than any known oral regimen [10],[13]–[15].

For resource-limited settings, a cost-effectiveness analysis of the induction antifungal treatment strategies will be valuable to guide stakeholders on optimal treatment regimens. Using current actual costs in Kampala, Uganda, we estimate the survival, cost, and cost per benefit associated with various treatment and care regimens for HIV-infected patients with CM.

## Methods

We conducted a decision analysis for HIV-infected adults with CM in resource-limited settings to estimate the incremental cost-effectiveness ratio (ICER) of the following induction treatment regimens: (1) fluconazole (800–1,200 mg/d) for 14 d; (2) fluconazole (1,200 mg/d) + 5FC (100 mg/kg/d) for 14 d; (3) amphotericin B (1.0 mg/kg/d) for 5–7 d ± fluconazole (1,200 mg/d) for 14 d; (4) amphotericin B (0.7–1.0 mg/kg/d) for 14 d; (5) amphotericin B (0.7–1.0 mg/kg/d) + fluconazole (800 mg/d) for 14 d; and (6) amphotericin B (0.7–1.0 mg/kg/d) + 5FC (100 mg/kg/d) for 14 d.

### Costs

Clinical and cost data were derived from current actual costs in Kampala, Uganda, as of May 31, 2012, in US dollars. We considered costs of medications, hospital care, diagnostics, laboratory monitoring, manometers, and personnel. Supply costs were current actual costs from a nonprofit medical wholesaler [16]. The amphotericin B deoxycholate wholesale cost in Kampala was US$5.92 per 50-mg dose, with a 2-wk course costing US$83.02. The cost of fluconazole was based on the Ugandan wholesale price of US$0.147 per 200-mg tablet. Pfizer's Diflucan Partnership Program was not considered in this analysis, as this donation program is not universally available. Given that 5FC is currently unavailable in most sub-Saharan African countries, the cost of 5FC was derived from an international wholesale cost of US$0.44 for a generic 500-mg tablet, as cited in [17]. The US cost of 5FC is 100-fold higher.

We assumed minimal laboratory monitoring for safety over 2 wk of hospitalization, in accordance with WHO guidelines [3], consisting of the following tests: three complete blood counts (US$6.20), five creatinine (US$5.00), and five electrolytes of sodium and potassium (US$4.75). One-week hospitalizations assumed one set of laboratory tests only. Two additional complete blood counts were added to the 5FC + fluconazole regimen to coincide with the laboratory monitoring described by Nussbaum et al. [11]. Laboratory test costs are actual average costs from the South African National Health Laboratory Service, the Liverpool Wellcome Trust Tropical Centre laboratory in Blantyre, Malawi, the Makerere University–Johns Hopkins University laboratory in Kampala, Uganda, and the Joint Clinical Research Centre laboratories in Uganda. A flat US$16.25 laboratory overhead fee was also assumed per patient to cover the cost of lab operation.

Regardless of treatment regimen, every patient was presumed to have one cryptococcal antigen (CRAG) test (US$9.80) and one cerebrospinal fluid (CSF) analysis (US$12.79) for the initial diagnostic lumbar puncture (LP). Each patient was presumed to have an average of three LPs during hospitalization—one diagnostic LP and two therapeutic LPs [18]. The LP cost included LP needles, collection tubes, lidocaine, syringes, sterile gloves, adhesive bandages, and gauze (US$2.42). The cost of using an imported manometer (US$8) for each LP performed was also included, although manometers are generally unavailable locally.

Other costs of hospital care included IV fluids, potassium and magnesium supplementation, IV lines and tubing, alcohol swabs, needles, syringes, and disposable gloves, totaling US$24.26 for 1 wk and US$48.44 for 2 wk. Each person was presumed to receive 1.5 L of IV fluids on admission [19], and thereafter only regimens containing amphotericin required IV fluids and electrolyte supplementation (e.g., potassium and magnesium). However, regimens without amphotericin were assumed to still use consumables such as alcohol swabs, syringes, and gloves (totaling US$1.89 per week). Hospital stay and hospital personnel costs were based on estimated figures from Mulago National Referral Hospital in Kampala, Uganda. Hospital stay cost was estimated to be US$3.92 per day per patient, plus personnel costs of full-time equivalents (FTE) per position considering the work days and/or nights per month (*n = *20) per position. We used the 2011–2012 government gross salaries of nurses (US$196/mo, 2.0 FTE), doctors (US$294/mo, 1.5 FTE), HIV counselors (US$196/mo, 1.0 FTE), and phlebotomists (US$154/mo, 1.0 FTE) divided by the daytime patient-to-staff ratio (20∶1) to calculate the personnel cost per patient per day.

Using the above cost estimates, we calculated the overall cost of each of the six induction treatment strategies. Oral regimens of fluconazole ± 5FC were presumed to require a 1-wk hospitalization with three LPs, without intensive electrolyte lab monitoring or daily IV fluids, whereas the amphotericin-based regimens were presumed to require hospitalization for the course of amphotericin, along with intensive lab monitoring, IV fluid provision, electrolyte supplementation, and three LPs.

Estimated actual cost per person for each induction treatment strategy ranged from US$154.17 for the fluconazole (1,200 mg) regimen to US$467.48 for amphotericin + 5FC (Table 1). Medication costs accounted for a small fraction of overall costs, ranging from 5.5% for fluconazole (800 mg) to 35.4% for amphotericin + 5FC. Including the Pfizer Diflucan Partnership Program decreased treatment costs by ≤8%.

**Table 1:** Input costs of cryptococcal meningitis induction therapy and medical care.

Induction Regimen	Duration of Induction	Costs					Total Cost of Care
		Medication	Three LPs w/Manometers	Hospital Supplies	Lab Costs	Personnel (Uganda)	
Fluconazole 800–1,200 mg	14 d	$8.23–$12.34	$53.85	$32.63	$36.95	$18.40^a^	$150.06–$154.17
5FC + fluconazole 1,200 mg	14 d	$85.98	$53.85	$32.63	$49.35	$20.74^a^	$242.55
Amphotericin + fluconazole 1,200 mg	7 d	$53.85	$53.85	$54.53	$36.95	$18.40^a^	$217.58
Amphotericin	14 d	$83.02	$53.85	$108.21	$107.35	$41.41	$393.84
Amphotericin + fluconazole 800 mg	14 d	$91.25	$53.85	$108.21	$107.35	$41.41	$402.07
Amphotericin + 5FC	14 d	$156.66	$53.85	$108.21	$107.35	$41.41	$467.48

LP includes initial diagnostic CSF analysis; 5FC dosed at 100 mg/kg/d; amphotericin B deoxycholate dosed at 0.7–1.0 mg/kg/d. Cost components displayed in Figure S1.

a Assumes 7 d of hospitalization, with additional phlebotomy for 5FC monitoring.

### Life Expectancy Assumptions

We performed a MeSH search of “cryptococcal meningitis” and “therapy,” and limited our findings to humans, adults, and English language results, to find 10-wk mortality data from trials and cohort studies evaluating treatment outcomes for CM induction regimens from 1996 onwards, i.e., in the antiretroviral therapy (ART) era. This search yielded 33 publications. After manually reviewing abstracts and references, 18 studies were included that presented mortality data for HIV-infected adults from resource-limited settings. We excluded studies that did not report 10-wk mortality, and CM studies conducted in the US or Europe. We limited our review to resource-constrained settings, as a cost-effectiveness analysis would be most pertinent and generalizable to these settings. Details on the included studies are provided in Table S1.

From these studies, pooled 10-wk mortality estimates were calculated for each of the treatment regimens. One-year mortality after CM treatment among those who survived the initial 10 wk was estimated at 11.2% (95% CI: 8.6%–14.1%); this estimate was derived from a weighted average from pooled South African CM cohorts (*n = *262), a Ugandan CM cohort (*n = *101), and a Thai CM cohort (*n = *277) [10],[20]–[22]. Mortality between 1 and 5 y after diagnosis was estimated at 11.5% based on the long-term follow-up of a previous Ugandan CM cohort [20],[23],[24], a Thai CM cohort on ART [21],[22], and a Ugandan cohort without CM initiating ART [25].

For life expectancy assumptions, those who died within 10 wk of CM diagnosis did not accrue any additional life years. For the 11.2% mortality between 10 wk and 1 y, the median survival was estimated as 18 wk based on pooled South African and Ugandan CM cohorts (*n = *363) [10],[20]. For those who died between 1 and 5 y after CM diagnosis, a life span of 3 y was assumed, such that the number of additional years lived beyond 1 y was the midpoint of the interval. The additional life expectancy of those who survived past 1 y was derived from age- and CD4-count-stratified life expectancies estimated from a Ugandan cohort without CM initiating ART [25]. This life expectancy was compared to the actual yearly mortality in the Ugandan CM cohort between 1 and 5 y [24] and was between that of two CD4 strata <50 and 50–100 CD4 cells/µl [25]; therefore, the average of the two CD4 strata was included in the model, with an estimated additional life expectancy of 18 y for those surviving at least 1 y (i.e., 19 y in total).

Quality-adjusted life years (QALYs) were calculated based on the Karnofsky functional status scale and expected life expectancy. In a prior Ugandan CM cohort [20], the mean Karnofsky score at 12 wk and 24 wk of ART was 95 (D. R. B., unpublished data). All life years beyond 10 wk were therefore adjusted for this 5% decrease in functional status experienced after CM treatment. We did not discount future benefits, as both the cost and survival benefit were immediately accrued, and postponement of treatment is not an option.

### Cost-Effectiveness Analysis

We compared induction regimens using the cost-effectiveness ratio as the primary end point. The ICER was calculated as the additional cost of a CM treatment strategy compared to fluconazole (1,200 mg) monotherapy, divided by the incremental improvement in QALYs [26]. To account for variations in costs and estimated outcomes, we performed a probabilistic sensitivity analysis incorporating the 95% CIs of the 10-wk, 1-y, and 5-y survival estimates and overall life expectancy estimates using TreeAge Pro 2012 (TreeAge Software). We also included in the sensitivity analysis a range of laboratory costs and medication costs based on the range of 2010 international reference costs for amphotericin (50-mg vial), median of US$5.27 (range: US$4.23–US$6.97 in Africa), and fluconazole (200-mg tablet), median of US$0.16 (range: US$0.14–US$0.19 in Africa) [27].

## Results

### Survival Benefit

From 18 relevant trials and cohorts evaluating outcomes of CM induction regimens in resource-limited settings, mean 10-wk mortality for each treatment regimen was calculated, and from this, 1-y mortality was projected (Table 2) [6]–[9],[11]–[13],[15],[20],[23],[24],[28]–[36]. In resource-limited settings, 10-wk mortality differed by approximately 30% between the most and least effective induction treatment regimens. The least effective treatment regimen was high-dose (800–1,200 mg/d) fluconazole monotherapy, with a 10-wk mortality of 54.9% (95% CI: 46.0%–63.5%). Pooled data from the four short-course amphotericin studies (amphotericin B at 1.0 mg/kg/d for 5–7 d ± fluconazole at 1,200 mg/d for 14 d) demonstrated a mean 10-wk mortality of 26.0% (95% CI: 18.6%–34.5%) [9],[13],[15],[31], comparable to all of the 2-wk amphotericin regimens (differences were non-significant).

**Table 2:** Estimated clinical outcomes by cryptococcal meningitis induction treatment regimen.

Induction Regimen	Duration of Induction	10-wk Mortality Mean	95% CI for 10-wk Mortality	1-y Mortality Mean (95% CI)	References
Fluconazole 800–1,200 mg	14 d	54.9% (73/133)	46.0%–63.5%	60% (54–66%)	[11],[12],[28],[29]
5FC + fluconazole 1,200 mg	14 d	43.5% (20/46)	28.9%–58.9%	50% (39–61%)	[11],[30] a
Amphotericin + fluconazole 1,200 mg	5–7 d^b^	26.0% (33/127)	18.6%–34.5%	34% (28–41%)	[9],[13],[15],[31]
Amphotericin	14 d	34.4% (128/372)	29.6%–39.5%	42% (38–51%)	[7],[8],[20],[24],[31]–[33]
Amphotericin + fluconazole 800 mg	14 d	30.0% (61/203)	23.8%–36.9%	38% (32–46%)	[6]–[8],[34]
Amphotericin + 5FC	14 d	26.8% (62/231)	21.2%–33.0%	35% (28–41%)	[6],[7],[34]–[36]

5FC dosed at 100 mg/kg/d; amphotericin B deoxycholate dosed at 0.7–1.0 mg/kg/d. Figure S2 displays the data.

a Mayanja-Kizza et al. used fluconazole doses of 200 mg/d and 5FC doses of 150 mg/kg/d [30].

b Muzoora et al. [13] used 5 d of amphotericin and Jackson et al. [15] used 7 d of amphotericin at 1.0 mg/kg/d with fluconazole at 1,200 mg/d, whereas 7 d of amphotericin was used by Bicanic et al. [9] (1.0 mg/kg/d) and Tansuphaswadikul et al. [31] (0.7 mg/kg/d).

Though fluconazole monotherapy alone is a more common option than amphotericin-based regimens because of its affordability, fluconazole was associated with greater than 2-fold higher 10-wk mortality (relative risk = 2.11; 95% CI: 1.52 to 2.94, *p*<0.001) and roughly 30% greater absolute mortality than short-course amphotericin. This translates into a number needed to treat of 3.5 (95% CI: 2.1 to 5.8) to save one additional life by using short-course amphotericin instead of fluconazole monotherapy. Short-course amphotericin also had better efficacy than the best oral regimen of fluconazole + 5FC (relative risk = 1.67, 95% CI: 1.07 to 2.60, *p* = 0.039).

### Base Case Cost-Effectiveness Analysis

**Figure 1:**
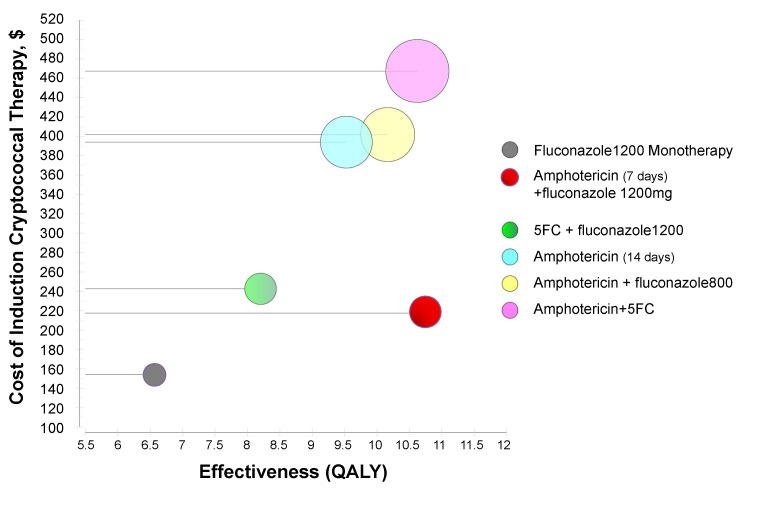
Cost effectiveness of cryptococcal induction therapies. This figure displays the cost of induction therapy for CM in resource-limited regions (in US dollars) versus the effectiveness as measured by QALYs saved per regimen. The radius of the circles represents the standard deviation of the cost estimate. Based on the existing outcome data, the short-course amphotericin (1 mg/d) + fluconazole (1,200 mg/d) regimen has similar effectiveness to but lower costs than traditional 2-wk amphotericin-based regimens. This short-course amphotericin + fluconazole regimen has marginally higher cost but significantly greater effectiveness than oral fluconazole-based therapies.

If CM patients are treated with a 14-d course of high-dose fluconazole (1,200 mg/d) alone, the average cost of care is US$154. Notably, fluconazole makes up only 8% of this cost. Estimated 1-y survival is 40% (95% CI: 34%–46%). Assuming an 18-y additional life span beyond 1 y among those survivors, the average patient gains an average estimated 6.55 QALYs, and the estimated cost-effectiveness ratio is US$23.53 per QALY gained (Table 3). In contrast, a 14-d course of amphotericin monotherapy costs US$394. One-year survival is 58%, with 9.52 QALYs gained. The cost-effectiveness ratio of 14 d of amphotericin alone is US$41.35 per QALY gained. Figure 1 displays the estimated QALYs gained versus the cost of cryptococcal induction therapy.

**Table 3:** Cost-effectiveness of six induction treatment strategies for cryptococcal meningitis in resource-limited settings.

Induction Regimen	Duration of Induction	Total Cost	Incremental Cost	1-y Survival Estimate	QALYs Gained^a^	Incremental Benefit (QALYs)	Cost-Effectiveness Ratio (US Dollars/QALY)	ICER (US Dollars/QALY)
Fluconazole 1,200 mg	14 d	$154.17	Reference	40.1%	6.55	Reference	$23.53	Reference
5FC + fluconazole 1,200 mg	14 d	$242.55	$88.38	50.2%	8.21	1.66	$29.55	$53.35
Amphotericin + fluconazole 1,200 mg	7 d	$217.58	$63.41	65.8%	10.75	4.20	$20.24	$15.11
Amphotericin	14 d	$393.84	$239.67	58.3%	9.52	2.97	$41.35	$80.60
Amphotericin + fluconazole 800 mg	14 d	$402.07	$247.90	62.2%	10.16	3.61	$39.58	$68.73
Amphotericin + 5FC	14 d	$467.48	$313.31	65.0%	10.62	4.07	$44.00	$76.93

a QALYs based on an estimated 18-y additional life expectancy with ART, after surviving 1 y of ART, based on the weighted average CD4 counts of persons with CM surviving 1 y on ART [25].

The short-course amphotericin regimen (7 d) with adjunctive fluconazole (1,200 mg/d) for 14 d costs US$218, slightly higher than the oral regimens, but with a much improved 1-y survival of 66% (95% CI: 60%–72%). The short-course 7-d amphotericin regimen had the most favorable cost-effectiveness ratio of US$20.24 per QALY, and the lowest ICER of all other treatment strategies. Compared to fluconazole monotherapy, short-course amphotericin provides an additional 4.2 QALYs at an incremental cost of US$15.11 per additional QALY. The oral regimen of 5FC + fluconazole was weakly dominated by the short-course amphotericin regimen, and the 2-wk amphotericin regimens were all strongly dominated by short-course amphotericin. That is, all of the 2-wk amphotericin regimens were more costly but no more effective than the short-course amphotericin strategy in resource-limited settings.

### Sensitivity Analysis

The most uncertain estimates in our analysis were the precision of 10-wk mortality and long-term life expectancy. To account for the potential range in mortality and life expectancy, we performed a sensitivity analysis. Table S2 provides the upper limit of the 95% CI for survival and the lower limit of the 95% CI for costs using a probabilistic sensitivity analysis. For short-course amphotericin, assuming a maximum 1-y survival of 72%, the cost effectiveness ratio is US$16.82, less than half that of the other amphotericin treatment strategies. As shown in Table S3, if one assumes the minimum 1-y survival of 59% for short-course amphotericin, the cost-effectiveness ratio is US$24.48, which remains lower than that of any of the alternative treatment strategies. Thus, using the 95% CI extremes of survival and costs did not change our finding that the short-course 7-d amphotericin + fluconazole regimen remains the most cost-effective treatment strategy.

### What is Cost-Effective?

Assuming a gross national income per capita of US$2,632 for the sub-Saharan African region in 2009 [37], all treatment strategies are highly cost-effective according to WHO definitions [26], with the short-course amphotericin + fluconazole regimen having the lowest cost-effectiveness ratio. Additionally, based on an estimated number needed to treat of 3.5 and an incremental cost of US$63 for the short-course amphotericin regimen above the cost of fluconazole monotherapy, the cost to save one additional life at 10-wk is US$220 using short-course amphotericin instead of fluconazole monotherapy.

## Discussion

Our analysis suggests that a 7-d course of amphotericin (1 mg/kg/d) with adjunctive high dose fluconazole (1,200 mg/d) for at least 2 wk is the most cost-effective cryptococcal induction treatment, with a cost-effectiveness ratio of US$20 per QALY gained. The short-course amphotericin regimen bridges the large cost disparity between the oral regimens and the 2-wk amphotericin regimens, and thus far seems to be similarly efficacious as the 2-wk amphotericin regimens in resource-constrained settings. Two-week amphotericin regimens are the most efficacious in high-income countries and are the first-line recommendation per WHO and US guidelines [3],[38],[39]. In low-income countries, however, 2 wk of amphotericin, along with hospitalization and monitoring of side effects, is unacceptably expensive and resource-intensive. Conversely, fluconazole therapy alone is a more common, accessible option given its affordability. Unfortunately, low cost does not make fluconazole monotherapy an optimal induction treatment regimen because other regimens, such as short-course amphotericin, appear to bridge the expanse between the efficacy extremes that currently exist. For example, fluconazole monotherapy has nearly 2-fold higher 10-wk mortality and 30% higher absolute mortality than short-course amphotericin. The number needed to treat is an incredibly low 3.5 persons treated using short-course amphotericin instead of fluconazole monotherapy to save one additional life. However, more evidence for the efficacy of short-course amphotericin with adjunctive fluconazole is needed. This is the only study to our knowledge that weighs the costs and benefits of care for CM in order to decipher which alternatives are most efficient from a policy standpoint.

While clearly requiring more infrastructure than an oral regimen alone, short-course amphotericin may be feasible for sites with limited amphotericin supplies, limited laboratory capacity, limited hospital bed space, and healthcare worker shortages. Short-course amphotericin with fluconazole (1,200 mg/d) can be safely given with potassium supplementation but without any routine laboratory monitoring [13],[15], as the cumulative amphotericin-related nephrotoxicity with urinary electrolyte wasting does not typically begin until after 5 d of amphotericin [40]–[42]. As amphotericin results in logarithmic CSF clearance of fungi [13],[14],[35], the maximal clinical and cost-effective benefit may be realized with short-course therapy, whereas beyond 1 wk, the further benefit of amphotericin may be countered by its toxicity, particularly in resource-limited settings. Head-to-head trials to better determine the optimal duration of amphotericin induction therapy are essential.

The limitations of this analysis stem mainly from the lack of high-quality evidence supporting the short-course amphotericin regimen. There are only four studies anywhere that have evaluated this strategy, with a total of 127 participants. Thus, due to lack of power, these studies were pooled for the purposes of this analysis. Each study used a slightly different amphotericin B deoxycholate regimen: Bicanic et al. [9] used 1.0 mg/kg/d alone for 7 d, Tansuphaswadikul et al. [31] used 0.7 mg/kg/d alone for 7 d, and Muzoora et al. [13] and Jackson et al. [15], respectively, used 5 and 7 d of amphotericin at 1.0 mg/kg/d with fluconazole at 1,200 mg daily in divided doses for 14 d [13],[15]. The moderately wide CIs pertaining to 10-wk mortality for all regimens, but especially short-course amphotericin (95% CI: 19%–35%), highlight the need for more clinical trials to evaluate the efficacy of this regimen. From the above-mentioned studies, one cannot draw evidence-based conclusions on superior efficacy for short-course (7-d) amphotericin with fluconazole; however, this regimen does appear to be the most cost-effective and clearly better than fluconazole monotherapy. Furthermore, there may be subtle differences between sites that limit precise cross-comparisons, such as stage of CM at presentation and management of elevated intracranial pressure. However, one illuminating experience is a series of three prospective studies in Mbarara, Uganda, between 2005 and 2012, in which the 10-wk mortality was 54% (31/57) with fluconazole (800–1,200 mg/d) monotherapy [12], 28% (8/29) with 5-d amphotericin + fluconazole (1,200 mg/d) [13], and 40% (16/40) with 14-d amphotericin + fluconazole (800 mg/d) in 2011–2012 (D. R. B., unpublished data from NCT01075152).

In addition, we have compared our cost–benefit analysis to the WHO-defined cost-effectiveness thresholds, which may be insufficient markers of cost-effectiveness. All of the treatment regimens analyzed here met the WHO criteria for being “highly cost effective.” However, clearly they are not equally efficacious regimens. In fact, our analysis suggests that within those “highly cost effective” regimens, some options are more efficacious than others, and merely following these WHO thresholds may lead policy-makers to institute a “cost-effective” strategy with lower efficacy, thereby ignoring superior regimens available with minimal additional cost. Additionally, from a policy perspective, we considered the cost-effectiveness only of CM induction treatment. Preventative health measures such as early ART (before AIDS) and targeted screening of asymptomatic cryptococcal antigenemia in individuals with a CD4 count<100 cells/µl are certainly more cost-effective to prevent disease than any of the aforementioned treatment options after cryptococcal disease has occurred [43]–[45].

As with any cost-effective analysis, what may be cost-effective may not be affordable for an individual patient or for a specific healthcare center, especially given that the Ugandan per capita expenditure on health in 2009 was approximately US$115 [46]. The purpose of this analysis is to inform stakeholders regarding where to invest resources on a national level. This analysis suggests that investing in building capacity for short-course amphotericin may be a cost-effective long-term strategy. Inclusion into the analysis of free fluconazole via the Diflucan Partnership Program, which is not available in all countries, does not change the ICER between fluconazole (1,200 mg/d) and short-course amphotericin + fluconazole (1,200 mg/d), as both regimens utilize the same amount of fluconazole. For stakeholder investment, the first resource to invest in is diagnostic capacity in order to diagnose cryptococcosis, such as the new point-of-care CRAG lateral flow assay (US$2 per test, Immy) [47], which is less expensive than traditional CRAG latex agglutination (US$13.50–US$16.25 in Malawi and Uganda), as the latex agglutination assay requires cold-chain shipping, which dramatically increases the real-world CRAG cost in Africa. Yet, many additional implementation science questions remain regarding how best to build capacity at rural healthcare facilities to administer short-course amphotericin. It may be most efficient to invest in larger referral centers that already have the capacity for amphotericin. Perhaps switching from a 14-d course to a 7-d course would free up healthcare workers, reduce burden on the laboratory, extend the benefit of amphotericin to more patients, and make more bed space available for other hospital admissions. If only larger centers are able to administer amphotericin, resources would have to be invested into triage and transport of patients with suspected CM at smaller, remote sites. The alternative is to develop the capacity to administer short-course amphotericin at these smaller rural centers, which would require a reliable supply and distribution of amphotericin, and further investment in healthcare workers for inpatient care of hospitalized patients.

One caveat is that control of intracranial pressure is equally important as the pharmaceutical regimen chosen for CM treatment [18],[48],[49]. Simply switching to 7 d of amphotericin will not mirror the reported outcomes without intracranial pressure control. The vast majority of studies included in this analysis controlled intracranial pressure through repeated therapeutic LPs, although the frequency of therapeutic LPs was not reported in each study. The assumption in this cost-effectiveness analysis was an average of three LPs (one diagnostic and two therapeutic) that would need to be performed to control intracranial pressure. The cost of manometers ranged from 5%–16% of the total cost of care. However, manometers, which support safe and accurate control of intracranial pressure, are generally unavailable in most healthcare centers and referral hospitals throughout Africa. Therefore, the scope of any capacity building must include supplies for safe LPs. In the absence of manometers, one approach could be to pre-screen with the point-of-care CRAG lateral flow assay (by fingerstick, plasma, serum, or urine) before LP [47], and then to either prioritize which patients to use a manometer on or empirically remove 20 ml of CSF with repeated therapeutic LP in 24–48 h in confirmed CM [18]. More operational research on the management of intracranial pressure in resource-limited settings is also urgently needed, including, for example, the use of high-quality IV tubing as a possible improvised manometer or surrogate noninvasive measures such as intraocular pressure [18],[50].

This analysis is certainly generalizable to sub-Saharan African countries with respect to estimates of 10-wk mortality and projected estimates of long-term survival, as all of the referenced papers were taken from similar resource-limited settings with a significant burden of CM. In sub-Saharan Africa, patients often present with more advanced cryptococcosis than in the US or Europe, as evidenced by longer duration of symptoms, higher CRAG titers, higher intracranial pressure, and greater proportion with altered mental status [5],[23]. Variation in the cost-effectiveness ratio within Africa is likely, as the actual costs of hospital personnel vary from country to country, and even within a single country, depending on the funding of the healthcare facility. Although astoundingly low, the personnel costs utilized in this analysis are correct and based on Ugandan national government public-sector salaries, where salaries are approximately 5-fold lower than in private-sector hospitals. When private-sector salaries are considered instead, the proportion of total costs accountable to personnel costs would increase from 8% to 32% for the short-course amphotericin regimen, with a resulting cost–benefit ratio of US$27/QALY and no change in the ICER versus fluconazole monotherapy. In South Africa, the total cost of caring for a patient with CM is estimated at US$2,883 [51]. While medications, supplies, and laboratory costs in South Africa are similar to our estimates, the specific regimen and cost components are unclear. Personnel costs are approximately 15-fold higher in South Africa and 4-fold higher in Rwanda than in the Uganda public sector, and healthcare worker to patient ratios are substantially better in South Africa, which may account for the difference in total cost of CM care. Still, there is no difference in the personnel necessary (and thereby the costs) between the fluconazole monotherapy and 7-d amphotericin + fluconazole regimens, thus the ICER remains unchanged even with varying personnel costs.

There are approximately 720,000 cases of CM annually in sub-Saharan Africa [1]. The WHO guidelines recommend amphotericin-based regimens, as they are clearly most effective. Many stakeholders instead rely on fluconazole monotherapy because it is more accessible, has lower upfront costs, and lacks the lab monitoring needs of amphotericin treatment, despite 25%–30% absolute higher mortality. We believe this is ineffective public health policy. Amphotericin should be moved from the “complementary list” to the “core list” in the *WHO Model List of Essential Medications* [52]. Our analysis suggests that using 7-d amphotericin (1 mg/kg/d) coupled with fungicidal doses of fluconazole (1,200 mg/d) for 2 wk appears to offer similar survival benefit but without the toxicity of 14 d of amphotericin in resource-constrained settings. Large-scale studies in resource-limited areas must be prioritized to determine efficacy, side effects, and risk of relapse with short-course amphotericin compared to more traditional 14-d amphotericin regimens. If the efficacy of short-course amphotericin is better determined, and survival is in fact ∼30% better than with fluconazole monotherapy, then moving to short-course amphotericin could save ∼150,000 lives annually (95% CI: 85,000 to 225,000) in sub-Saharan Africa, at a cost of US$220 per life saved.

## Supporting Information

Alternative Language Abstract S1 Translation of the abstract into Spanish by Dr. Jose Debes.
(DOCX)

Alternative Language Abstract S2 Translation of the abstract into Russian by Dr. Irina Vlasova-St. Louis.
(DOC)

Alternative Language Abstract S3 Translation of the abstract into Portuguese by Dr. Jose E. Vidal.
(DOC)

Alternative Language Abstract S4 Translation of the abstract into Japanese by Dr. Kosuke Yasukawa.
(DOC)

Alternative Language Abstract S5 Translation of the abstract into French by Drs. Anali Conesa Botella, Angela Loyse, and Sonia Helmy.
(DOC)

Figure S1 Input costs of cryptococcal meningitis induction therapy and medical care.
(TIF)

Figure S2 Mortality after cryptococcal meningitis by treatment regimen.
(TIFF)

Table S1 Summary of included cryptococcal treatment studies.
(XLSX)

Table S2 Maximum of all assumptions for cost-effectiveness of six induction treatment strategies for cryptococcal meningitis in resource-limited settings.
(DOC)

Table S3 Minimum of all assumptions for cost-effectiveness of six induction treatment strategies for cryptococcal meningitis in resource-limited settings.
(DOC)

## Abbreviations

5FC: flucytosine
ART: antiretroviral therapy
CM: cryptococcal meningitis
CRAG: cryptococcal antigen
CSF: cerebrospinal fluid
FTE: full-time equivalents
ICER: incremental cost-effectiveness ratio
IV: intravenous
LP: lumbar puncture
QALY: quality-adjusted life year
WHO: World Health Organization

## References

[1] Park BJ, Wannwmuehler KA, Marston BJ, Govender N, Pappas PG, et al (2009) Estimation of the current global burden of cryptococcal meningitis among persons living with HIV/AIDS. AIDS 23: 525–530. 19182676 10.1097/QAD.0b013e328322ffac

[2] Liechty CA, Solberg P, Were W, Ekwaru JP, Ransom RL, et al (2007) Asymptomatic serum cryptococcal antigenemia and early mortality during antiretroviral therapy in rural Uganda. Trop Med Int Health 12: 929–935. 17697087 10.1111/j.1365-3156.2007.01874.x

[3] World Health Organization (2011) Rapid advice: diagnosis, prevention and management of cryptococcal disease in HIV-infected adults, adolescents and children. Geneva: World Health Organization. Available: http://whqlibdoc.who.int/publications/2011/9789241502979_eng.pdf. Accessed 31 May 2012. 26110194

[4] Dromer F, Bernede-Bauduin C, Guillemot D, Lortholary O (2008) Major role for amphotericin B-flucytosine combination in severe cryptococcosis. PLoS ONE 3: e2870 doi:10.1371/journal.pone.0002870. 18682846 10.1371/journal.pone.0002870 PMC2483933

[5] van der Horst CM, Saag MS, Cloud GA, Hamill RJ, Graybill JR, et al (1997) Treatment of cryptococcal meningitis associated with the acquired immunodeficiency syndrome. National Institute of Allergy and Infectious Diseases Mycoses Study Group and AIDS Clinical Trials Group. N Engl J Med 337: 15–21. 9203426 10.1056/NEJM199707033370103

[6] Day JN, Chau TTH, Dung NT, Mai PP, Phu NH, et al. (2011) Combination antifungal therapy for HIV associated cryptococcal meningitis [abstract]. 51st Interscience Conference on Antimicrobial Agents and Chemotherapy; 17–20 September 2011; Chicago, Illinois, United States.

[7] Brouwer AE, Rajanuwong A, Chierakul W, Griffin GE, Larsen RA, et al (2004) Combination antifungal therapies for HIV-associated cryptococcal meningitis: a randomised trial. Lancet 363: 1764–1767. 15172774 10.1016/S0140-6736(04)16301-0

[8] Pappas PG, Chetchotisakd P, Larsen RA, Manosuthi W, Morris MI, et al (2009) A phase II randomized trial of amphotericin B alone or combined with fluconazole in the treatment of HIV-associated cryptococcal meningitis. Clin Infect Dis 48: 1775–1783. 19441980 10.1086/599112

[9] Bicanic T, Meintjes G, Wood R, Hayes M, Rebe K, et al (2007) Fungal burden, early fungicidal activity, and outcome in cryptococcal meningitis in antiretroviral-naive or antiretroviral-experienced patients treated with amphotericin B or fluconazole. Clin Infect Dis 45: 76–80. 17554704 10.1086/518607

[10] Bicanic T, Jarvis J, Loyse A, Jackson A, Muzoora C, et al. (2011) Determinants of acute outcome and long-term survival in HIV-associated cryptococcal meningitis: results from a combined cohort of 523 patients [abstract]. 18th Conference on Retroviruses and Opportunistic Infections; 27 Feb–2 Mar 2011; Boston, Massachusetts, United States.

[11] Nussbaum JC, Jackson A, Namarika D, Phulusa J, Kenala J, et al (2010) Combination flucytosine and high-dose fluconazole compared with fluconazole monotherapy for the treatment of cryptococcal meningitis: a randomized trial in Malawi. Clin Infect Dis 50: 338–344. 20038244 10.1086/649861 PMC2805957

[12] Longley N, Muzoora C, Taseera K, Mwesigye J, Rwebembera J, et al (2008) Dose response effect of high-dose fluconazole for HIV-associated cryptococcal meningitis in southwestern Uganda. Clin Infect Dis 47: 1556–1561. 18990067 10.1086/593194

[13] Muzoora CK, Kabanda T, Ortu G, Ssentamu J, Hearn P, et al (2012) Short course amphotericin B with high dose fluconazole for HIV-associated cryptococcal meningitis. J Infect 64: 76–81. 22079502 10.1016/j.jinf.2011.10.014

[14] Bicanic T, Muzoora C, Brouwer AE, Meintjes G, Longley N, et al (2009) Independent association between rate of clearance of infection and clinical outcome of HIV associated cryptococcal meningitis: analysis of a combined cohort of 262 patients. Clin Infect Dis 49: 702–709. 19613840 10.1086/604716 PMC2965403

[15] Jackson A, Nussbaum J, Phulusa J, Namarika D, Chikasema M, et al (2012) A phase II randomised controlled trial adding oral flucytosine to high dose fluconazole, with short-course amphotericin B, for cryptococcal meningitis in Malawi. AIDS 26: 1363–1370. 22526517 10.1097/QAD.0b013e328354b419 PMC3776948

[16] Joint Medical Store (2011) Catalogue. Available: http://www.jms.co.ug/uploads/catalogue.pdf. Accessed 31 May 2012.

[17] Bartlett JG, Auwaerter PG, Pham PA (2012) Johns Hopkins ABX guide: diagnosis and treatment of infectious disease, 3rd edition. Baltimore(Maryland): Jones and Bartlett Learning.

[18] Bicanic T, Brouwer AE, Meintjes G, Rebe K, Limmathurotsakul D, et al (2009) Relationship of cerebrospinal fluid pressure, fungal burden and outcome in patients with cryptococcal meningitis undergoing serial lumbar punctures. AIDS 23: 701–706. 19279443 10.1097/QAD.0b013e32832605fe

[19] Jacob ST, Moore CC, Banura P, Pinkerton R, Meya D, et al (2009) Severe sepsis in two Ugandan hospitals: a prospective observational study of management and outcomes in a predominantly HIV-1 infected population. PLoS ONE 4: e7782 doi:10.1371/journal.pone.0007782. 19907656 10.1371/journal.pone.0007782 PMC2771355

[20] Boulware DR, Meya DB, Bergemann TL, Wiesner DL, Rhein J, et al (2010) Clinical features and serum biomarkers in HIV immune reconstitution inflammatory syndrome after cryptococcal meningitis: a prospective cohort study. PLoS Med 7: e1000384 doi:10.1371/journal.pmed.1000384. 21253011 10.1371/journal.pmed.1000384 PMC3014618

[21] Manosuthi W, Chottanapund S, Sungkanuparph S (2008) Mortality rate of early versus deferred initiation of antiretroviral therapy in HIV-1-infected patients with cryptococcal meningitis. J Acquir Immune Defic Syndr 48: 508–509. 18614933 10.1097/QAI.0b013e318168573d

[22] Chottanapund S, Singhasivanon P, Kaewkungwal J, Chamroonswasdi K, Manosuthi W (2007) Survival time of HIV-infected patients with cryptococcal meningitis. J Med Assoc Thai 90: 2104–2111. 18041430

[23] Kambugu A, Meya DB, Rhein J, O'Brien M, Janoff EN, et al (2008) Outcomes of cryptococcal meningitis in Uganda before and after the availability of highly active antiretroviral therapy. Clin Infect Dis 46: 1694–1701. 18433339 10.1086/587667 PMC2593910

[24] Butler E, Meya DB, Boulware DR (2011) Early treatment of Cryptococcal antigenemia improves long-term survival as compared to treatment of symptomatic cryptococcal meningitis in persons with HIV/AIDS [abstract]. 2011 Global Health Conference; 13–15 Nov 2011; Montreal, Canada.

[25] Mills EJ, Bakanda C, Birungi J, Chan K, Ford N, et al (2011) Life expectancy of persons receiving combination antiretroviral therapy in low-income countries: a cohort analysis from Uganda. Ann Intern Med 155: 209–216. 21768555 10.7326/0003-4819-155-4-201108160-00358

[26] World Health Organization (2003) Making choices in health: WHO guide to cost-effectiveness analysis. Geneva: World Health Organization.

[27] Management Sciences for Health (2010) International drug price indicator guide: drug price search—search year: 2010. Available: http://erc.msh.org/dmpguide. Accessed 31 May 2012.

[28] Kisenge PR, Hawkins AT, Maro VP, McHele JP, Swai NS, et al (2007) Low CD4 count plus coma predicts cryptococcal meningitis in Tanzania. BMC Infect Dis 7: 39. 17493266 10.1186/1471-2334-7-39 PMC1876460

[29] Wajanga BM, Kalluvya S, Downs JA, Johnson WD, Fitzgerald DW, et al (2011) Universal screening of Tanzanian HIV-infected adult inpatients with the serum cryptococcal antigen to improve diagnosis and reduce mortality: an operational study. J Int AIDS Soc 14: 48. 21988905 10.1186/1758-2652-14-48 PMC3197468

[30] Mayanja-Kizza H, Oishi K, Mitarai S, Yamashita H, Nalongo K, et al (1998) Combination therapy with fluconazole and flucytosine for cryptococcal meningitis in Ugandan patients with AIDS. Clin Infect Dis 26: 1362–1366. 9636863 10.1086/516372

[31] Tansuphaswadikul S, Maek-a-Nantawat W, Phonrat B, Boonpokbn L, Mctm AG, et al (2006) Comparison of one week with two week regimens of amphotericin B both followed by fluconazole in the treatment of cryptococcal meningitis among AIDS patients. J Med Assoc Thai 89: 1677–1685. 17128844

[32] Boulware DR, Bonham SC, Meya DB, Wiesner DL, Park GS, et al (2010) Paucity of initial cerebrospinal fluid inflammation in cryptococcal meningitis is associated with subsequent immune reconstitution inflammatory syndrome. J Infect Dis 202: 962–970. 20677939 10.1086/655785 PMC2924457

[33] Chang CC, Dorasamy AA, Elliott JH, Naranbhai V, Gosnell BI, et al. (2012) HIV-infected patients with cryptococcal meningitis who attain CSF sterility pre-cART commencement experience improved outcomes in the first 24 weeks [abstract]. 19th Conference on Retroviruses and Opportunistic Infections; 5–8 Mar 2012; Seattle, Washington, United States.

[34] Loyse A, Wilson D, Meintjes G, Jarvis JN, Bicanic T, et al (2012) Comparison of the early fungicidal activity of high-dose fluconazole, voriconazole, and flucytosine as second-line drugs given in combination with amphotericin B for the treatment of HIV-associated cryptococcal meningitis. Clin Infect Dis 54: 121–128. 22052885 10.1093/cid/cir745

[35] Bicanic T, Wood R, Meintjes G, Rebe K, Brouwer A, et al (2008) High-dose amphotericin B with flucytosine for the treatment of cryptococcal meningitis in HIV-infected patients: a randomized trial. Clin Infect Dis 47: 123–130. 18505387 10.1086/588792

[36] Jarvis JN, Meintjes G, Rebe K, Williams GN, Bicanic T, et al (2012) Adjunctive interferon-gamma immunotherapy for the treatment of HIV-associated cryptococcal meningitis: a randomized controlled trial. AIDS 26: 1105–1113. 22421244 10.1097/QAD.0b013e3283536a93 PMC3640254

[37] World Health Organization (2011) World health statistics 2011. Available: http://www.who.int/gho/publications/world_health_statistics/EN_WHS2011_Full.pdf. Accessed 31 May 2012.

[38] Kaplan JE, Benson C, Holmes KH, Brooks JT, Pau A, et al (2009) Guidelines for prevention and treatment of opportunistic infections in HIV-infected adults and adolescents: recommendations from CDC, the National Institutes of Health, and the HIV Medicine Association of the Infectious Diseases Society of America. MMWR Recomm Rep 58: 1–207. 19357635

[39] Perfect JR, Dismukes WE, Dromer F, Goldman DL, Graybill JR, et al (2010) Clinical practice guidelines for the management of cryptococcal disease: 2010 update by the Infectious Diseases Society of America. Clin Infect Dis 50: 291–322. 20047480 10.1086/649858 PMC5826644

[40] Bahr N, Rolfes MAR, Musubire A, Nabeta H, Lo M, et al. (2011) The impact of routine electrolyte supplementation during amphotericin induction therapy in resource-limited settings [abstract]. 8th International Conference on Cryptococcus and Cryptococcosis; 1–5 May 2011; Charleston, South Carolina, United States.

[41] Girmenia C, Gentile G, Micozzi A, Martino P (2001) Nephrotoxicity of amphotericin B desoxycholate. Clin Infect Dis 33: 915–916. 11512101 10.1086/322716

[42] Mayer J, Doubek M, Vorlicek J (1999) Must we really fear toxicity of conventional amphotericin B in oncological patients? Support Care Cancer 7: 51–55. 9926976 10.1007/s005200050224

[43] Meya DB, Manabe YC, Castelnuovo B, Cook BA, Elbireer AM, et al (2010) Cost-effectiveness of serum cryptococcal antigen screening to prevent deaths among HIV-infected persons with a CD4+ cell count < or = 100 cells/microL who start HIV therapy in resource-limited settings. Clin Infect Dis 51: 448–455. 20597693 10.1086/655143 PMC2946373

[44] Jarvis JN, Lawn SD, Vogt M, Bangani N, Wood R, et al (2009) Screening for cryptococcal antigenemia in patients accessing an antiretroviral treatment program in South Africa. Clin Infect Dis 48: 856–862. 19222372 10.1086/597262 PMC2875173

[45] Rajasingham R, Meya DB, Boulware DR (2012) Integrating cryptococcal antigen screening and pre-emptive treatment into routine HIV care. J Acquir Immune Defic Syndr 59: 85–91 doi:10.1097/QAI.1090b1013e31824c31837e. 10.1097/QAI.0b013e31824c837e PMC3311156 22410867

[46] World Health Organization (2011) Data from the Global Health Observatory: country health profile—Uganda. Available: http://www.who.int/gho/countries/uga.pdf. Accessed 31 May 2012.

[47] Jarvis JN, Percival A, Bauman S, Pelfrey J, Meintjes G, et al (2011) Evaluation of a novel point-of-care cryptococcal antigen test on serum, plasma, and urine from patients with HIV-associated cryptococcal meningitis. Clin Infect Dis 53: 1019–1023. 21940419 10.1093/cid/cir613 PMC3193830

[48] Pappas PG (2005) Managing cryptococcal meningitis is about handling the pressure. Clin Infect Dis 40: 480–482. 15668875 10.1086/427222

[49] Shoham S, Cover C, Donegan N, Fulnecky E, Kumar P (2005) Cryptococcus neoformans meningitis at 2 hospitals in Washington, D.C.: adherence of health care providers to published practice guidelines for the management of cryptococcal disease. Clin Infect Dis 40: 477–479. 15668874 10.1086/427213

[50] Manosuthi W, Sungkanuparph S, Chottanapund S, Tansuphaswadikul S, Chimsuntorn S, et al (2008) Temporary external lumbar drainage for reducing elevated intracranial pressure in HIV-infected patients with cryptococcal meningitis. Int J STD AIDS 19: 268–271. 18482948 10.1258/ijsa.2007.007286

[51] Jarvis JN, Lawn SD, Wood R, Harrison TS (2010) Cryptococcal antigen screening for patients initiating antiretroviral therapy: time for action. Clin Infect Dis 51: 1463–1465. 10.1086/657405 21082878

[52] World Health Organization (2011) WHO model list of essential medicines, 17th edition. Available: http://www.who.int/medicines/publications/essentialmedicines. Accessed 31 May 2012.

