# Supplementary material for: Cryptococcal Meningitis Treatment Strategies in Resource-Limited Settings: A Cost-Effectiveness Analysis
Source: PLoS Med. 2012 Sep 25;9(9):e1001316. doi: 10.1371/journal.pmed.1001316 (PMC3463510; doi:10.1371/journal.pmed.1001316)
Supplement: Alternative Language Abstract S1 — Translation of the abstract into Spanish by Dr. Jose Debes. (DOCX) [file pmed.1001316.s001.docx]

Spanish: Translation of the abstract into Spanish by Dr. Jose Debes.

Abstract:

**Introduccion**: La meningitis por criptococo (CM) es la causa mas comun de meningitis en Africa. LA OMS recomienda un tratamiento con anfotericina de 14 dias como terapia de induccion. Sin embargo, este tratamiento no es practico en areas con bajos recursos debido a los costos y el monitoreo necesario. Desarrolamos un analysis de costo-efectividad como guia para un tratamiendo optimo de CM en areas de bajos recursos.

**Metodos y Resultados**: Realizamos un analisis de decision para estimar el radio incremental de costo-efectividad (ICER) de 6 regimenes de induccion para CM: monoterapia con fluconazol 800-1200 mg/dia, fluconazol + flucitosina (5FC), corto plazo de anfotericina (7 dias) + fluconazole, 14 dias de anfotericina sola, anfotericina + fluconazol y anfotericina + 5FC. Analizamos los costos de salud actuales del 2012 en Uganda para medicamentos, personal, insumos y costos aproximados de laboratorios de 3 paises. Un estudio sistematico de tratamientos de criptococo en areas de bajo recursos resumio los resultados de sobrevida de 10 semanas. Modelamos la sobrevida a un año basados en resultados de meningitis por criptococo de Uganda, Sudafrica y Tailandia, y sobrevida de mas de un año en resultados de Uganda y Tailandia. Los años de vida ajustados por calidad (QALY) se determinaron y fueron usados para calcular el costo efectividad de ICER.

El costo hospitalario vario desde US$ 154 para monoterapia con fluconazol hasta US$ 467 para 14 dias de anfotericina + 5FU. Basados en 18 estudios investigando resultados de pacientes HIV con CM en areas de bajos recursos, la sobrevida media estimada a una año fue un 40% menor para monoterapia con fluconazol. El radio de costo-efectividad vario de US$20 a US$44 por QALY. Los regimenes basados en anfotericina fueron los de mas alto costo, pero tuvieron la mayor sobrevida. Anfotericina de corto plazo (1mg/Kg/dia por 7 dias) con fluconazol (1200 mg/dia por 14 dias) tuvo la mayor sobrevida a un año (66%) y la mayor costo-efectividad a US$20.24/QALY, con un ICER de US$15.11 por QALY adicional sobre la monoterapia de fluconazol. La principal limitacion de este trabajo es el caracter grupal del estudio sistematico, con una falta de comparacion directa de datos y resultados.

**Conclusiones**: Corto plazo (7 dias) de induccion con anfotericina sumado a altas dosis de fluconazole (1200mg/dia) tiene una gran costo-efectividad basado en criterios de la OMS y podria ser una inversion apropiada para politicas de salud que buscan resultados costo-efectivos. Mas estudios comparativos son necesarios en esta enfermedad desatendida.
