# Supplementary material for: Cryptococcal Meningitis Treatment Strategies in Resource-Limited Settings: A Cost-Effectiveness Analysis
Source: PLoS Med. 2012 Sep 25;9(9):e1001316. doi: 10.1371/journal.pmed.1001316 (PMC3463510; doi:10.1371/journal.pmed.1001316)
Supplement: Alternative Language Abstract S2 — Translation of the abstract into Russian by Dr. Irina Vlasova-St. Louis. (DOC) [file pmed.1001316.s002.doc]

Russian: Translation of the abstract into Russian by Dr. Irina Vlasova-St. Louis.

Сравнение экономической эффективности различных режимов лечения криптококкового менингита

**Введение:** Криптококковый менингит – самая распространенная форма менингитов в центральной Африке. ВОЗ рекомендует 14-дневный вступительный курс амфотерицина В, хотя это не практично в районах с ограниченными ресурсами (по причине высокой цены препарата и невозможности интенсивного отслеживания пациентов). Анализ проведен c целью составить руководящие принципы для оптимальной терапии криптоккового менингита (КМ) при ограниченных средствах медицинского обслуживания.

**Методы и результаты:** Мы провели анализ отношения возрастания медицинских издержек к изменению эффективности лечения (здесь индекс ICER) шести медикаментозных курсов КМ: монотерапия флуконазола 800-1200 мг в день; флуконазол + флуцитозин (5FC), короткий курс амфотерицина В (7 дней) + флуконазол; монотерапия амфотерицина В (14 дней); амфотерицин В + флуконазол; и амфотерицин В + 5FC.

Мы подсчитали и сравнили среднюю стоимость мед препаратов, персонала и лабораторного обслуживания за 2012 год в трех странах (только в районах с ограниченными ресурсами). Основываясь на данных по 10-недельной выживаемости пациентов, мы смоделировали годовую выживаемость после криптококкового менингита исходя из данных полученных из Южной Африки, Юганды и Тайланда, а так же показатель выживаемости более года, после КМ, - из Юганды и Тайланда. 'Индекс отражающий качественно прожитые годы' (здесь QALY) был определен и затем использован для подсчета коэффициента эффективности лечения (стоимость/эффективность) и индекса ICER. Стоимость госпитального обслуживания варьировалась от $154 (монотерапия флуконазола) до $467 (амфотерицина В + флуцитозин, 14 дней). Результаты 18 исследований на ВИЧ-инфицированных больных с криптококковым менингитом в бедных районах показали самую низкую (40%) выживаемость при монотерапии флуконазола. Коэффициент ‘стоимость/эффективность' варьировалась от $20 до $44 на каждый QALY. В общем, хотя полная стоимость курса амфотерицина В была выше, однако, и выживаемость пациентов также увеличилась. Короткий курс амфотерицина В (1мг/кг/день, 7 дней) с флуконазолом (1200 мг/день, 14 дней) сопровождался самой высокой годовой выживаемостью (66%) и наиболее благоприятным коэффициентом эффективности лечения ($20,24/ QALY), что на $15,11/QALY выше,чем при монотерапии флуконазола. Наши результаты получены выборочным путем, из нескольких независимых клинических испытаний, однако не прямым сравнением подгрупп лечения в одном исследовании.

**Заключение:** Короткий семидневный курс амфотерицина В скомбинированный с высокой дозой (1200 мг/день) флуконазола является самым экономичным и эффективным (по предложенным критериям ВОЗ). Курс заслуживает внимания экспертов, финансирующих разработку экономичных и эффективных принципов лечения. Дополнительные клинические испытания необходимы для разработки протоколов лечения этого тропического заболевания, часто оставленного без внимания.
