# Supplementary material for: Cryptococcal Meningitis Treatment Strategies in Resource-Limited Settings: A Cost-Effectiveness Analysis
Source: PLoS Med. 2012 Sep 25;9(9):e1001316. doi: 10.1371/journal.pmed.1001316 (PMC3463510; doi:10.1371/journal.pmed.1001316)
Supplement: Alternative Language Abstract S3 — Translation of the abstract into Portuguese by Dr. Jose E. Vidal. (DOC) [file pmed.1001316.s003.doc]

Portuguese: Translation of the abstract into Portuguese by Dr. Jose E Vidal.

**Resumo**

**Introdução:** Meningite criptocócica (MC) é a causa mais comum de meningite na África. As guias de OMS recomendam esquemas de indução baseados em 14 dias de anfotericina; porém, isso é muito complicado em diversos cenários com recursos limitados, devido ao custo e necessidade de monitoramento intensivo. Foi realizada uma análise de custo-efetividade para guiar a otimização do tratamento da MC em contextos com recursos limitados.

**Métodos e Resultados:** Conduzimos uma análise de decisões para estimar a razão de custo-efetividade incremental (RCEI) de seis esquemas de indução para MC, incluindo: fluconazol 800-1200mg/dia em monoterapia, fluconazol + flucitosina (5FC), curso curto de anfotericina (7-dias) + fluconazol, 14 dias de anfotericina em monoterapia, anfotericina + fluconazol, e anfotericina + 5FC. Avaliamos os custos em saúde na Uganda em 2012, para medicações, materiais, pessoal, e promedios de custos laboratoriais em três países. Uma revisão sistemática de ensaios terapêuticos para criptococose, em contextos de recursos limitados, resumiram desfechos de sobrevida em 10 semanas. Estruturamos um ano de sobrevida baseados em dados de sobrevida de MC na África do Sul, Uganda e Tailândia, e sobrevida além de um ano, com dados de Uganda e Tailândia. Foram determinadas qualidades de vida ajustadas por anos (QVAA) e usadas para calcular a relação custo-efetividade e RCEI.

O custo dos cuidados hospitalares variou de $154 para monoterapia com fluconazol até $467 1para 14 dias de anfotericina + 5FC. Baseados em 18 estudos que investigaram desfechos de pacientes infectados pelo HIV com MC, em contextos de recursos limitados, a media estimada de um ano de sobrevida foi menor em 40% para a monoterapia com fluconazol. A relação custo-efetividade variou de $20 até $44 por QVAA. Ao todo, esquemas baseados em anfotericina tiveram maiores custos, mas melhores sobrevidas. O curso curto de anfotericina (1mg/kg/dia por 7 dias) con fluconazol (1200mg/dia por14 dias) teve a melhor sobrevida em um ano (66%) e a relação custo-efetividade mais favorável, com $20.24/QVAA, e um RCEI de $15.11, por QVAA adicional, sobre a monoterapia com fluconazol. A principal limitação deste estudo é a natureza concentrada de uma revisão sistemática, com limitados dados prognósticos provenientes de comparações diretas.

**Conclusões:** Um curso curto (7 dias) de tratamento de indução com anfotericina associada a doses altas de fluconazol (1200mg/dia) é “muito custo efetivo” pelos critérios da OMS e pode ser um investimento desejável para gestores que procuram desfechos clínicos custo-efetivos. Mais ensaios clínicos cabeça a cabeça são necessários sobre essa doença tropical negligenciada.
