# Supplementary material for: Cryptococcal Meningitis Treatment Strategies in Resource-Limited Settings: A Cost-Effectiveness Analysis
Source: PLoS Med. 2012 Sep 25;9(9):e1001316. doi: 10.1371/journal.pmed.1001316 (PMC3463510; doi:10.1371/journal.pmed.1001316)
Supplement: Alternative Language Abstract S4 — Translation of the abstract into Japanese by Dr. Kosuke Yasukawa. (DOC) [file pmed.1001316.s004.doc]

Japanese: Translation of the abstract into Japanese by Dr. Kosuke Yasukawa.

**抄録**

**背景**：クリプトコッカス髄膜炎（CM：cryptococcal meningitis）は、アフリカにおける髄膜炎で最も頻度が高い。WHOのガイドラインでは、14日間のアムホテリシンをベースとした導入療法が推奨されている。しかし、費用と厳密なモニタリングが必要なことから医療資源の限られた状況では実用的ではない。資源の限られた状況での最も適切なCM治療について関係者の参考になるように、費用効果分析を行った。

**方法と結果**：

CMの6つの導入療法レジメンについての増分費用効果比（ICER：incremental cost-effectiveness ratio）を推定するために決定分析を行った。分析を行ったレジメンは、フルコナゾール(800-1200mg/日)単独治療、フルコナゾール＋フルシトシン(5FC)、短期間(7日間)のアムホテリシン＋フルコナゾール、14日間のアムホテリシン単独治療、14日間のアムホテリシン＋フルコナゾール、14日間のアムホテリシン＋5FCである。薬剤・物資の費用および人件費については2012年におけるウガンダの医療費を用い、また検査費については３カ国の平均値を用い計算した。途上国で行われたCM治療についての臨床試験のシステマティック・レビューを行い、10週生存率をもとめた。1年生存率については南アフリカ、ウガンダ、タイでのCMの臨床結果を、1年以降の生存についてはウガンダとタイの結果を基にモデル化した。質調整生存年(QALY: Quality-adjusted life years)を決め、費用効果比及びICERを計算した。

　　　院内ケアの費用は、フルコナゾール単独治療の154ドルから、14日間のアムホテリシン＋5FCの467ドルの範囲であった。医療資源の限られた状況におけるHIV感染者のCMの転帰について調査した18個の試験によると、推定1年生存率はフルコナゾール単独治療で最も低く40%であった。費用効果比は、20ドル/QALYから44ドル/QALYであった。概して、アムホテリシンを含むレジメンは、より費用がかかるが、生存率は高かった。短期間のアムホテリシン(1mg/kg/日 を7日間)にフルコナゾール(1200mg/日を14日間)を併用した治療の1年生存率(66%)が最も良く、費用効果比も20.24ドル/QALYと最も高かった。また、フルコナゾール単剤療法とのICERはQALYあたり15.11ドル であった。本研究の主な限界点は、治療について直接比較した臨床結果が少なく、システマティック・レビューによって複数の研究結果を統合していることである。

**結論：**短期間(7日間)のアムホテリシンによる導入療法に高容量(1200mg/日)のフルコナゾールを併用した治療は、WHOの基準によれば「非常に費用効果が高い」といえ、費用効果の高い臨床結果を求める政策担当者にとっては価値のある投資かもしれない。この顧みられない熱帯病についてより多くの直接比較試験が必要である。
