# Supplementary material for: Cryptococcal Meningitis Treatment Strategies in Resource-Limited Settings: A Cost-Effectiveness Analysis
Source: PLoS Med. 2012 Sep 25;9(9):e1001316. doi: 10.1371/journal.pmed.1001316 (PMC3463510; doi:10.1371/journal.pmed.1001316)
Supplement: Alternative Language Abstract S5 — Translation of the abstract into French by Drs. Anali Conesa Botella, Angela Loyse, and Sonia Helmy. (DOC) [file pmed.1001316.s005.doc]

French: Translation of the abstract into French by Drs. Anali Conesa Botella, Angela Loyse, and Sonia Helmy.

**Abstract**

**Introduction:**

La méningite à cryptococque (MC) est la plus fréquente cause de méningite en Afrique. L’OMS recommande 14 jours de traitement à base d’amphotéricine B. Cependant, ce traitement est incommode pour de nombreux pays à ressources limités, dû au coût et aux besoins de suivi intensif. Une analyse qualité-prix a été effectuée pour guider les dirigeants de médecine publique conçernant le traitement optimal de la MC dans des contextes cliniques à ressources limitées.

**Méthodes et Résultats:**

Nous avons effectué une analyse de décision pour estimer l’incrément du rapport de coût-efficacité (IRCE) des six régimes d’induction pour la MC incluant: monothérapie avec la fluconazole 800-1200mg/jour, fluconazole + flucytosine (5FC), traitement court à l’amphotéricine (7 jours) + fluconazole, 14 jours d’amphotéricine seule, amphotéricine + fluconazole, et amphotéricine+5FC.

Nous avons calculé les coûts réels pour 2012 de soins de santé en Ouganda, pour les médicaments, l’approvisionnement, le personnel, et la moyenne des coûts de laboratoires dans 3 pays. Nous avons completé une revue systématique des essais clinique de traitement de la méningite à cryptococque dans des régions à resources limités pour déterminer la survie à 10 semaines.

Nous avons créé un modèle de survie à 1 ans sur la base des données de méningite à cryptococque provenant d’Afrique du Sud, d’Ouganda et de Thaïlande ; et la survie au-delà d’un an basée sur les données provenant de l’Ouganda et de la Thaïlande. Les années de vie gagnées pondérées par leur qualité (quality-adjusted life years : QALY) ont été calculées et utilisées pour l’évaluation du rapport coût-efficacité et le IRCE.

Le coût des soins hospitaliers variait de 154$ pour la monothérapie au fluconazole, à 467$ pour 14 jours d’amphotéricine+5FC. Basé sur 18 études sur les conséquences d’une MC chez des patients infectés par le VIH dans des pays à ressources limitées, l’estimation de la moyenne de survie à un an était la plus basse, de l’ordre de 40%, pour la monothérapie au fluconazole.

Le rapport coût-efficacité variant de 20$ à 44$ par QALY. En général, les régimes à base d’amphotéricine étaient plus chers mais donnaient une survie plus élevée. Un traitement court d’amphotericine (1mg/kg/jour pendant 7 jours) avec du fluconazole (1200mg/jour pendant 14 jours) donnait la meilleure survie à 1 an (66%), et avait le rapport coût-efficacité le plus favorable à 20,24$/QALY, avec un IRCE de 15,11$ par QALY additionnel, par rapport à la monothérapie au fluconazole. La principale limitation de cette étude est la nature groupée de la revue systématique, avec peu de résultats pouvant être directement comparés.

**Conclusions:**

Un traitement court (7 jours) d’induction à l’amphotéricine, associé à une haute dose de fluconazole (1200mg/jour) présente un « très bon rapport coût-efficacité » selon les critères de l’OMS, et pourrait représenter un choix de traitement intéressant pour les autorités politiques cherchant des résultats cliniques favorables à prix abordables. Plus d’études cliniques competitives sont nécessaires pour cette maladie tropicale négligée.
