# Supplementary figures and images for: Cryptococcal Meningitis Treatment Strategies in Resource-Limited Settings: A Cost-Effectiveness Analysis
Source: PLoS Med. 2012 Sep 25;9(9):e1001316. doi: 10.1371/journal.pmed.1001316 (PMC3463510; doi:10.1371/journal.pmed.1001316)

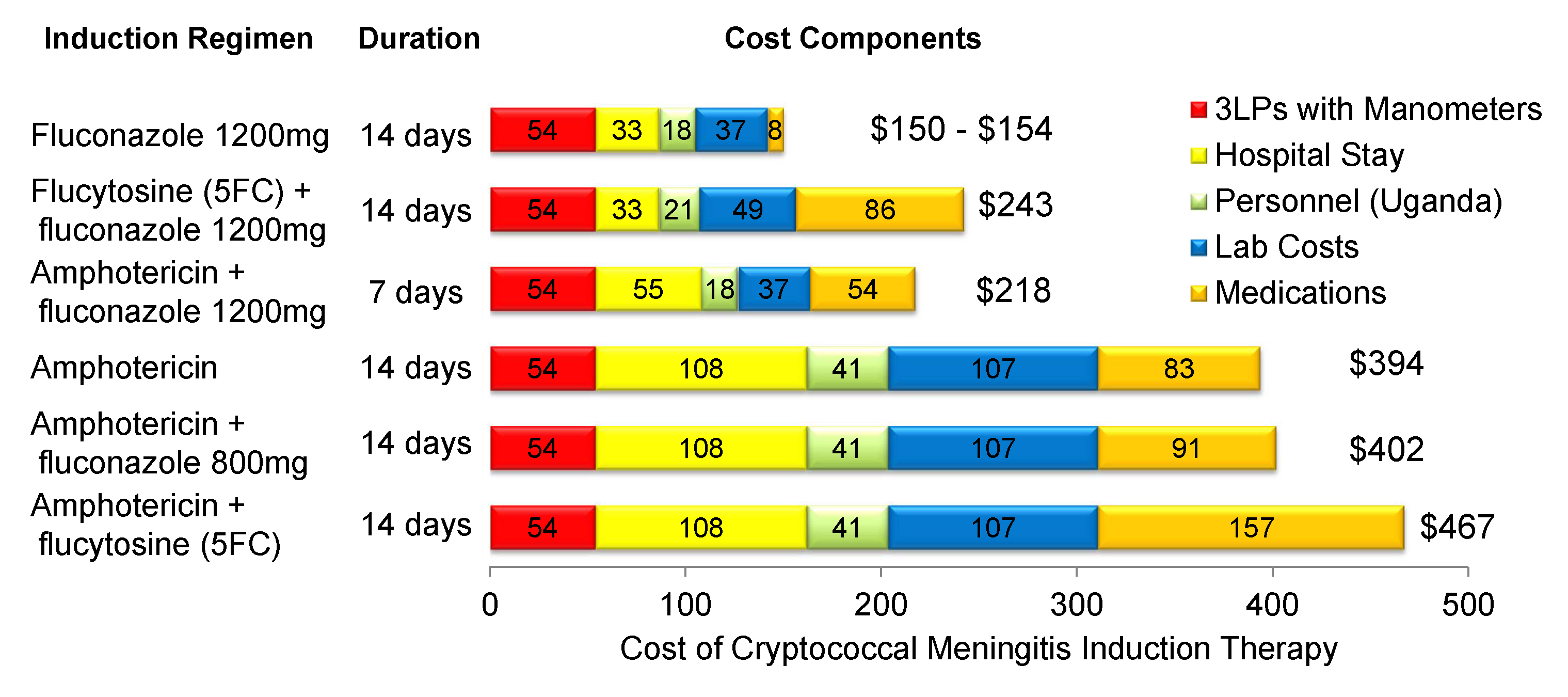

Supplement: Figure S1 — Input costs of cryptococcal meningitis induction therapy and medical care. (TIF) [file pmed.1001316.s006.tif]

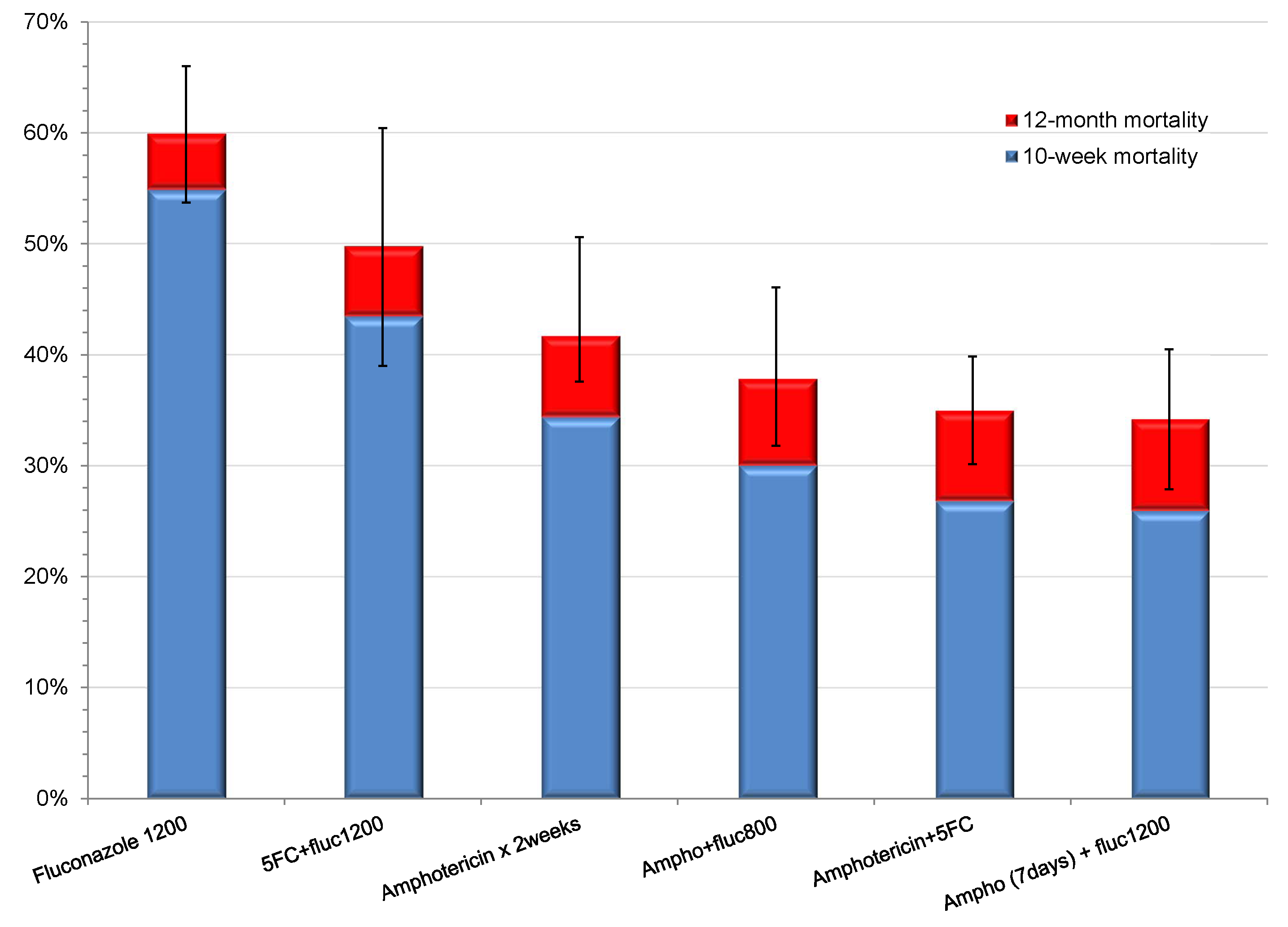

Supplement: Figure S2 — Mortality after cryptococcal meningitis by treatment regimen. (TIFF) [file pmed.1001316.s007.tiff]
