# Supplementary material for: Cryptococcal Meningitis Treatment Strategies in Resource-Limited Settings: A Cost-Effectiveness Analysis
Source: PLoS Med. 2012 Sep 25;9(9):e1001316. doi: 10.1371/journal.pmed.1001316 (PMC3463510; doi:10.1371/journal.pmed.1001316)
Supplement: Table S2 — Maximum of all assumptions for cost-effectiveness of six induction treatment strategies for cryptococcal meningitis in resource-limited settings. (DOC) [file pmed.1001316.s009.doc]

**Table S2: Maximum of all assumptions for cost-effectiveness of six induction treatment strategies for cryptococcal meningitis in resource-limited settings.**

| **Induction Regimen** | **Duration of Induction** | **Low Cost**  **of Total Care** | **1-year survival** | **QALYs gained** | **CE ratio ($/QALY)** | **ICER ($/increased QALYs)** |
| --- | --- | --- | --- | --- | --- | --- |
| **Fluconazole 1200mg** | 14 days | $143.17 | 46.3% | 7.72 | $18.55 | Reference |
| **Flucytosine (5FC) + fluconazole 1200mg** | 14 days | $225.93 | 61.0% | 10.12 | $22.33 | $34.48 |
| **Amphotericin + fluconazole 1200mg** | 7 days | $204.50 | 72.1% | 12.16 | $16.82 | $13.81 |
| **Amphotericin** | 14 days | $319.13 | 62.4% | 10.59 | $30.14 | $61.31 |
| **Amphotericin + fluconazole 800mg** | 14 days | $327.38 | 68.2% | 11.40 | $28.72 | $50.06 |
| **Amphotericin + flucytosine (5FC )** | 14 days | $393.09 | 69.9% | 11.85 | $33.17 | $60.51 |

Survival estimates and QALY based on upper 95% confidence interval bound via probabilistic sensitivity analysis. Costs are estimated at the lower 95% confidence interval bound incorporating range of medication and lab costs.

QALY=quality adjusted life years; CE = cost-effectiveness; QALYs based on an estimated 18-year life additional expectancy with ART, after surviving one year of ART, based on weighted CD4 average, based on weighted average of CD4 of persons with CM surviving one year on ART [25]
