# Supplementary material for: Cryptococcal Meningitis Treatment Strategies in Resource-Limited Settings: A Cost-Effectiveness Analysis
Source: PLoS Med. 2012 Sep 25;9(9):e1001316. doi: 10.1371/journal.pmed.1001316 (PMC3463510; doi:10.1371/journal.pmed.1001316)
Supplement: Table S3 — Minimum of all assumptions for cost-effectiveness of six induction treatment strategies for cryptococcal meningitis in resource-limited settings. (DOC) [file pmed.1001316.s010.doc]

**Table S3: Minimum** **of all assumptions for cost-effectiveness of six induction treatment strategies for cryptococcal meningitis in resource-limited settings.**

| **Induction Regimen** | **Duration of Induction** | **High Cost of Total Care** | | **1-year survival** | | **QALYs gained** | | **CE ratio ($/QALY)** | | **ICER ($/increased QALYs)** | |
| --- | --- | --- | --- | --- | --- | --- | --- | --- | --- | --- | --- |
| **Fluconazole 1200mg** | 14 days | $165.2 | 34.0% | | 5.44 | | $30.37 | | Reference | |  |
| **Flucytosine (5FC)+ fluconazole 1200mg** | 14 days | $259.32 | 39.5% | | 6.37 | | $40.71 | | $101.18 | |  |
| **Amphotericin + fluconazole 1200mg** | 7 days | $230.38 | 59.5% | | 9.41 | | $24.48 | | $16.41 | |  |
| **Amphotericin** | 14 days | $467.39 | 49.4% | | 8.51 | | $54.92 | | $98.43 | |  |
| **Amphotericin + fluconazole 800mg** | 14 days | $476.62 | 53.9% | | 8.98 | | $53.08 | | $87.97 | |  |
| **Amphotericin + flucytosine (5FC)** | 14 days | $542.33 | 60.2% | | 9.45 | | $57.39 | | $94.04 | |  |

Survival estimates and QALY based on lower 95% confidence interval bound via probabilistic sensitivity analysis. Costs are estimated at the higher 95% confidence interval bound incorporating range of medication and lab costs.

QALY=quality adjusted life years; CE = cost-effectiveness; ICER = incremental cost effectiveness ratio. QALYs based on an estimated 18-year life additional expectancy with ART after surviving one year of ART based on weighted average CD4 of persons with CM surviving one year on ART [25]
